# Evaluating TIMP-2 and IGFBP-7 as a predictive tool for kidney injury in ureteropelvic junction obstruction

**DOI:** 10.1590/S1677-5538.IBJU.2021.0340

**Published:** 2021-01-13

**Authors:** Marcos Figueiredo Mello, José de Bessa, Sabrina T. Reis, Enzo Yagi Kondo, Luis Yu, Francisco Tibor Dénes, Roberto Iglesias Lopes

**Affiliations:** 1 Unidade de Urologia Pediátrica da Divisão de Urologia Hospital das Clínicas da Faculdade de Medicina da Universidade de São Paulo São Paulo SP Brasil Unidade de Urologia Pediátrica da Divisão de Urologia do Hospital das Clínicas da Faculdade de Medicina da Universidade de São Paulo, São Paulo, SP, Brasil; 2 Universidade Estadual de Feira de Santana Feira de Santana Divisão de Urologia Bahia BA Brasil Divisão de Urologia, Universidade Estadual de Feira de Santana, Feira de Santana, Bahia, BA, Brasil; 3 Universidade de São Paulo Faculdade de Medicina Divisão de Urologia São Paulo SP Brasil Laboratório de Investigação Médica (LIM55), Divisão de Urologia, Faculdade de Medicina da Universidade de São Paulo, São Paulo, SP, Brasil; 4 Universidade de São Paulo Hospital das Clínicas São Paulo SP Brasil Divisão de Nefrologia, Hospital das Clínicas, Faculdade de Medicina da Universidade de São Paulo, São Paulo, SP, Brasil

**Keywords:** Cakut [Supplementary Concept], Urinary Tract, Child, Biomarkers

## Abstract

A major challenge in the management of ureteropelvic junction obstruction (UPJO) is the selection of patients who would benefit from surgical treatment. Tissue inhibitor metalloproteinase-2 (TIMP-2) and insulin-like growth factor-binding protein 7 (IGFBP7) indicate renal cell stress and are associated with cell cycle arrest. The [TIMP-2] [IGFBP7] ratio (Nephrocheck®) has been recently applied in patients in intensive care units patients to predict the development of acute kidney injury. In this study, we evaluated the performance of these biomarkers performance to distinguishing obstructive hydronephrosis (HN) from non-obstructive HN.

**Materials and Methods:**

Consecutive patients with UPJO were enrolled in this study. Urinary [TIMP-2] [IGFBP7] and clinical characteristics (hydronephrosis grade, differential renal function, and drainage half-time) were measured in the following groups: 26 children with obstructive HN at initial diagnosis (group 1A) and after six months of dismembered pyeloplasty (group 1B); 22 children with non-obstructive HN (group 2), and 26 children without any urinary tract condition, as the control group (group 3).

**Results:**

Comparing the initial samples, [TIMP-2] [IGFBP7] had higher levels in the HN groups and lower levels in the control group; however, no difference was observed between the HN groups (obstructive vs. non-obstructive). After six months of follow-up, patients who underwent dismembered pyeloplasty showed stability in the urinary concentration of [TIMP-2] [IGFBP7]. All patients with [TIMP-2] [IGFBP7] higher than 1.0 (ng/mL)^2^/1000 had diffuse cortical atrophy on ultrasonography.

**Conclusions:**

We showed that urinary levels of urinary [TIMP-2] [IGFBP7] are higher in children with HN than controls. Nephrocheck® is not reliable in predicting the need for surgical intervention for pediatric patients with UPJO.

## INTRODUCTION

NephroCheck® (Astute Medical, San Diego, CA, USA) is an immunoassay test that measures the urinary concentrations of two cell-cycle arrest biomarkers, tissue inhibitor metalloproteinase-2 (TIMP-2) and insulin-like growth factor-binding protein 7 (IGFBP7), and provides a quantitative risk index that combines them into a single numerical ratio\([TIMP-2] [IGFBP7]/1000), which is a reliable predictor of acute kidney injury (AKI) in critically ill patients, the results of which can be expected within 1 hour of sample collection (1). In comparison with serum creatinine and the urinary output, it has the advantage of detecting renal damage even in the absence of glomerular filtration rate (GFR) alterations (2).

Ureteropelvic junction obstruction (UPJO) particularly represents a challenge regarding treatment indications and its follow-up. The main issue is that UPJO is a spectral disease, where in not all cases of hydronephrosis (HN) represent a kidney-damaging state, and decision-making involves choosing between the options for binary treatment: surgical correction or surveillance.

There has been controversy regarding the indications of surgical intervention in asymptomatic patients. Some authors have proposed initial nonoperative management along with intensive imaging protocols with surgical intervention primarily based on decreasing ipsilateral differential renal function or increasing the drainage half-time (3). However, in the era of patient-oriented medicine, several studies have focused on the identification of ultrasonography findings or serum and urinary biomarkers for managing UPJO in children.

Morphological changes associated with urinary obstruction include tubular dilation, and atrophy, thickening of the basement membrane, and interstitial fibrosis. TIMP-2 and IGFBP7 have a molecular weight of approximately 24 kDa and 29 kDa respectively (4). Both are expressed and secreted by renal tubular cells and involved in G1 cell cycle arrest during the early phases of cellular stress or injury caused by various insults (e.g., sepsis, ischemia, oxidative stress, and toxins) (5).

When exposed to cellular stress or injury, renal tubular cells may produce and release TIMP-2 and IGFBP7. TIMP-2 stimulates p27 expression and IGFBP7 directly increases the expression of p53 and p21. These proteins block the effect of cyclin-dependent protein kinase complexes (CyclD-CDK4 and CyclE-CDK2) on cell cycle promotion, resulting in transient G1 cell cycle arrest, thereby providing cells with an opportunity to repair DNA damage and regain function. This process occurs during early cellular stress and may help cells maintain energy balance and prevent further DNA damage and division (6). However, sustained cell cycle arrest results in a senescent cell phenotype and leads to fibrosis. Therefore, cell cycle arrest is not only associated with an increased risk of AKI but may also serve as a link between AKI and renal fibrosis (7, 8).

Our hypothesis is that cell cycle arrest biomarkers (TIMP-2 and IGFBP7) have higher urinary levels in patients with UPJO who require surgical intervention (associated with interstitial fibrosis) compared to those in patients elected for surveillance.

## MATERIAL AND METHODS

This was a case-control prospective study performed from September 2016 to June 2019 at our institution (research ethics board approval #62235816.8.0000.0068). Children with congenital HN caused by UPJO diagnosed at the pediatric unit of the Department of Urology, and healthy children without underlying pathologies were enrolled from the childcare outpatient clinic. All subjects were aged between 1 month and 18 years. All caregivers of the children were interviewed and provided informed consent for the children to participate in the study.

The diagnosis of HN without ureteral dilatation was primarily confirmed using renal ultrasonography (US). The degree of HN was graded according to the Society for Fetal Urology (SFU) classification and the alternative grading score proposed by Onen [Onen's alternative grading system (AGS)] on renal US (9).

**Figure 1:**
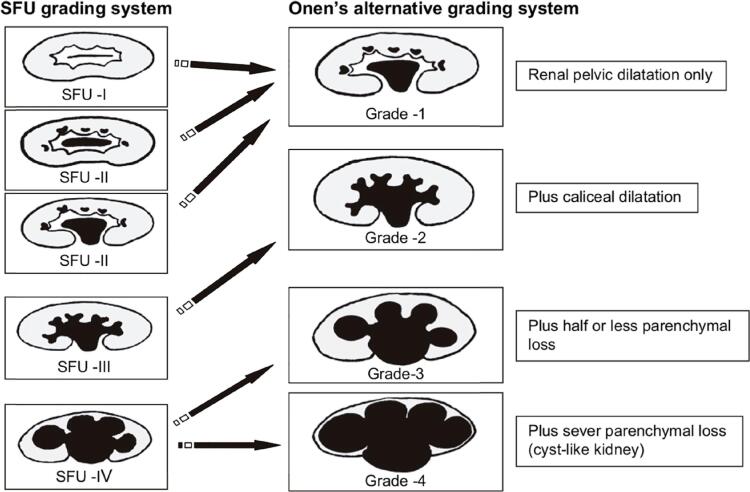
Comparison of SFU hydronephrosis grading system and Onen's AGS.

Irreversible renal damage may occur particularly in patients with severe HN; Onen's AGS has the advantage of accurately indicate this condition by dividing SFU-4 into two different groups. The criteria for correlation between the two systems are as follows: SFU-1 (pelvic dilatation alone) and SFU-2 (pelvic dilatation with minimal calyceal dilatation) is considered AGS-1, SFU-3 (calyceal dilation without parenchymal thickness) is considered AGS-2, and SFU-4 (calyceal dilation with parenchymal thickness) is classified as either AGS-3 when the renal parenchymal thickness represents half or less of the contralateral normal kidney or AGS-4 if there is severe parenchymal loss (cyst-like kidney) (10) (Figure-1).

UPJO was diagnosed as HN without ipsilateral ureteral dilatation on US; if vesicoureteral reflux was suspected, voiding cystourethrography was performed. Subsequently, a radioisotope renal scan was performed in all cases to confirm the diagnosis. At our institution, as technetium-99m mercaptoacetyltriglycine renography is not available, both diuretic renography [diethylenetriaminepentaacetate (DTPA) radionuclide renal scan] and static renography [dimercaptosuccinic acid (DMSA) radionuclide renal scans) were performed. DMSA radionuclide renal scan was interpreted as differential renal functions (DRFs): a DRF of the affected dilated kidney of <40% was considered abnormal. DTPA radionuclide renal scan curves were classified according to Lee (10): pattern IV of Lee was considered obstructed.

The indications for surgical intervention (pyeloplasty) were based on the available guidelines (11–14): symptomatic obstruction, impaired DRF (<40%), decrease of >10% in renal function in subsequent investigations, poor drainage function after the administration of diuretics, increasing APD, or worsening HN to SFU grades III and IV on US from the current proposed indications for surgical intervention (11).

The exclusion criteria included associated anomalies, such as vesicoureteral reflux, ureterovesical junction obstruction, and posterior urethral valve obstruction; bilateral HN; previous operation of the urinary system; other deformations in the external genital organs, lower part of the ureter, bladder, and urethra; urinary stones; and neurogenic bladder dysfunction.

The patients were categorized into three groups based on the clinical and imaging findings, including the APD, DRF, and drainage curve on the radioisotope renal scan: group 1 (obstructive HN), group 2 (non-obstructive HN) and control group.

The patients were followed up for at least 6 months; they underwent regular clinic visits, US, and radionuclide renal scans. The clinical work-up included the analysis of medical charts to determine age, sex, laterality, grade of HN, anteroposterior pelvic diameter, age at the time of diagnosis, method of treatment, measurement of blood pressure, and physical examination results. The biochemical work-up included the determination of serum creatinine levels.

In group 1 (obstructive HN), voided urinary samples were collected twice: preoperatively (baseline evaluation) before surgical repair for UPJO (examination A) and 6 months postoperatively (examination B). In group 2 (non-obstructive HN) and the control group, urinary samples were collected only once at baseline. Collection was based on the patient's age and ability to spontaneously void, with a preference for midstream urinary samples (whenever possible). Urinary culture was obligatory to exclude active urinary infections and avoid interference with the studied urinary markers.

Urine was aseptically collected, mixed and centrifuged (3000rpm, 10 min), and stored in samples frozen at - 80°C. After thawing, the urine creatinine concentration was determined (measured using the Jaffe reaction), and measurements of urinary IGBP-7 and TIMP-2 (using microsphere-based LuminexÒ technology, as described below) were performed.

Urine specimens were prepared for analysis in 96-well plates using the 4-cytokine Milliplex MAP Human Premixed Multi-Analyte Kit LXSAHM-04-1KIT (BiotechneÒ, Minneapolis, MN) following the manufacturer's protocols. Analytes were quantified using a Magpix analytical test instrument, which utilizes xMAP technology, multiple analyte profiling (Luminex Corp., Austin, TX), and xPONENT 4.2 software (Luminex). Concentrations of cytokines (pg/mL) were determined based on the fit of a standard curve for mean fluorescence intensity (pg/mL).

### Statistical Analysis

Statistical analysis was performed using IBM SPSS Statistics for Windows, Version 17.0 (SPSS, Chicago, IL, USA) and the R platform (v.3.2.5). Continuous variables are shown as median±standard error of the mean, and categorical variables are presented as frequencies. A comparison of the demographic, clinical, imaging, and urinary variables was performed. For homogeneous groups, a one-way analysis of variance (ANOVA) was conducted. For nonparametric groups, the Mann-Whitney U test or Kruskal-Wallis test was performed. The influence of the time decrease in urinary markers was also evaluated using ANOVA. Statistical significance was set at p <0.05. Finally, receiver-operating characteristic (ROC) curves were generated to determine the optimal cut-off point (using Youden's index) for the studied urinary markers in UPJO.

## RESULTS

This study enrolled 74 patients. Group 1 (obstructive HN) comprised 26 children (15 boys, 11 girls) with a median age of 4.9±4.7 years. Group 2 (non-obstructive HN) included 22 children (13 boys, 9 girls; median age 6.9±4.8 years). Group 3 (control group) consisted of 26 healthy children (17 boys, 9 girls; median age 6.1±3.4 years).

The demographic characteristics and clinical data for each group are presented in Table-1. All patients had a normal estimated GFR >90mL/min/1:73m2 calculated using the Schwartz formula (15). In group 2 (non-obstructive HN), unilateral HN was more common on the left side (72%), and in group 1 (obstructive HN), the laterality was even. As expected, there was statistically significant difference between the groups regarding the symptoms, degree of HN on the SFU grading scale based on US, DRF based on DMSA scans, and pattern of renal scan curves based on DTPA scans (Table-1).

**Table 1:** Demographic and clinical characteristics parameters of the patient and control groups showed as average, minimum and maximum.

Parameters	Group 1 (obstructive HN)	Group 2 (non-obstructive HN)	Control	p
Age (years) a	4.9 (0-17)	6.9 (0-18)	6.1 (0-15)	0.077
Sex b (male/female)	15/11	13/9	17/9	0.835
Laterality b (right/left)	13/13	6/16	NE	0.109
Clinical concern b	13	0	NE	0.001
APD (mm) c	31.2 (16-65)	19.7 (7-40)	NE	0.001
SFU grade IV b	16 (62%)	0	NE	0.001
Onen`s AGS IV b	8 (31%)	0	NE	0.001
Split renal function c	37.7 (11-57)	48.4 (40-55)	NE	0.005
Lee curve IV b	26 (100%)	0	NE	0.001

APD = anterior-posterior diameter of affected renal pelvis; HN = hydronephrosis; NE = not evaluated.

a Kruskal Wallis Test

b Pearson Chi-Square Test

c Mann-Whitney Test

**Figure 2:**
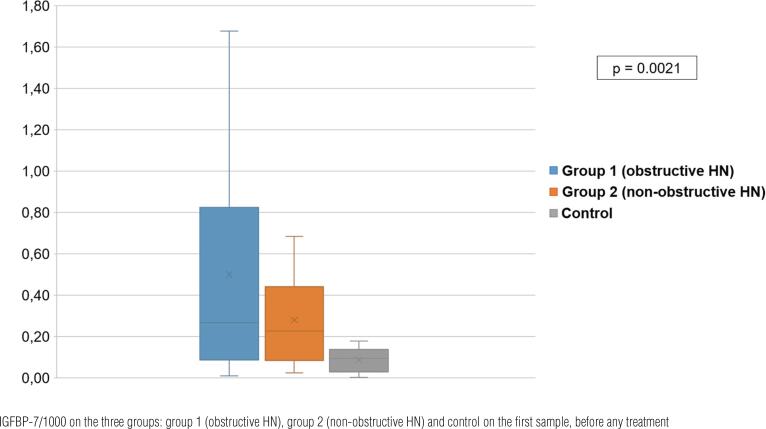
Figure shows concentrations of TIMP-2.

Baseline evaluations of [TIMP-2] [IGFBP7]/1000 revealed the following concentrations: Group 1 (obstructive HN), 0.29 (0.10-0.85) (ng/mL)^2^/1000; Group 2 (non-obstructive HN), 0.23 (0.09-0.42) (ng/mL)^2^/1000, and control group, 0.12 (0.03-0.18) (ng/mL)^2^/1000. [TIMP-2] [IGFBP-7] had different concentrations in the three groups, with higher values according to the severity of obstruction (groups 1 and 2 vs. controls, p=0.0021). However, higher levels of [TIMP-2] [IGFBP-7]/1000 in group 1 (obstructive HN) did not exhibit statistically significant differences when compared to those in group 2 (non-obstructive HN) (Figure-2).

**Figure 3:**
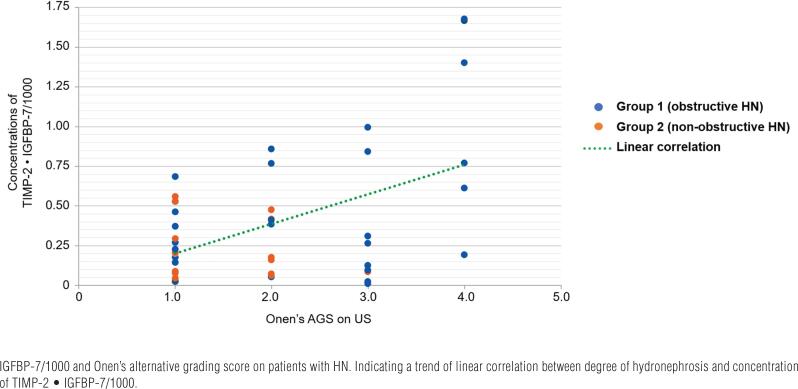
The figure shows the correlation between first samples concentrations, before any treatment, of TIMP-2.

ROC curve analysis showed a disappointing diagnostic profile for the detection of obstructive HN for [TIMP-2] [IGFBP7]/1000 [area under the curve (AUC) of 0.5]. All patients with [TIMP-2] [IGFBP7]/1000 greater than 1.0 had diffuse cortical atrophy on US (Onen's AGS 4) (Figure-3).

The follow-up analysis of urine concentrations of the [TIMP-2] [IGFBP7] comparisons in group 1 (obstructive HN) between the preoperative sample (0.29; 0.10-0.85) and postoperative follow-up sample (0.29; 0.16-0.65) showed a trend of stability in these values (p=0.335) (Table-2).

**Table 2:** All data of 74 patients.

	Group	Age (years)	Sex	Laterality	Clinical concern	APD (mm)	SFU grade score	Onen`s AGS	Split renal function	Lee curve	[TIMP-2] • [IGFBP7] /1000
1	1	11.42	Female	Right	Yes	18	2	1	27,7	4	0,27
2	1	10.93	Male	Left	Yes	16	3	2	57	4	0,77
3	1	8.98	Male	Left	Yes	22	2	1	37	4	0,53
4	1	8.13	Male	Left	Yes	20	2	1	42	4	0,03
5	1	7.48	Female	Left	Yes	19	4	3	26	4	0,99
6	1	1.37	Male	Left	No	29	4	3	35	4	0,84
7	1	0.42	Female	Left	No	50	4	4	40	4	1,67
8	1	2.59	Male	Right	No	36	3	2	22	4	0,16
9	1	8.31	Female	Right	Yes	30	4	4	25	4	0,19
10	1	8.19	Male	Right	No	24	4	3	51,5	4	0,13
11	1	1.52	Male	Right	No	36	4	3	48	4	0,31
12	1	1.70	Male	Left	Yes	32	4	4	47	4	66,19
13	1	4.00	Male	Left	Yes	61	4	4	12	4	0,77
14	1	1.24	Female	Right	No	16	2	1	22	4	0,18
15	1	2.24	Male	Right	Yes	20	3	2	48	4	0,05
16	1	0.45	Male	Left	Yes	28	3	2	43	4	1,68
17	1	2.17	Male	Right	No	21	3	2	55	4	0,06
18	1	0.36	Male	Right	No	21	3	2	45	4	0,86
19	1	1.09	Female	Left	No	20	4	4	11	4	1,40
20	1	0.48	Female	Left	No	37	4	4	23	4	3,27
21	1	16.87	Female	Right	Yes	36	4	3	29	4	0,26
22	1	9.02	Male	Left	Yes	62	4	3	52	4	0,08
23	1	2.08	Female	Right	No	23	4	3	34	4	0,01
24	1	12.13	Female	Right	Yes	43	4	3	46	4	0,02
25	1	1.38	Male	Left	No	65	4	4	43	4	0,61
26	1	1.88	Female	Right	No	27	4	3	48	4	0,09
27	2	6.57	Male	Left	No	28	2	1	47	2	0,45
28	2	0.77	Female	Left	No	22	2	1	50	3	0,53
29	2	0.53	Female	Left	No	21	2	1	51	2	0,20
30	2	4.20	Male	Left	No	14	1	1	51	2	0,68
31	2	6.45	Male	Left	No	20	1	1	55	2	0,14
32	2	0.15	Male	Left	Yes	11	1	1	52	2	0,56
33	2	1.94	Male	Left	Yes	18	1	1	51	1	0,29
34	2	0.52	Female	Right	No	7	1	1	47	2	0,37
35	2	10.68	Male	Right	No	18	1	1	50	1	0,08
36	2	7.91	Female	Left	No	17	3	2	47	2	0,17
37	2	4.99	Female	Right	No	26	3	2	41	2	0,42
38	2	7.40	Male	Left	No	40	3	2	49	3	0,48
39	2	0.85	Female	Right	No	19	3	2	48	1	0,41
40	2	8.11	Male	Right	No	18	3	2	46	3	0,38
41	2	17.98	Male	Left	No	32	1	1	47	2	0,09
42	2	0.68	Female	Left	No	9	2	1	40	3	0,21
43	2	0.46	Female	Right	Yes	16	3	2	47	4	0,07
44	2	1.65	Male	Left	No	18	1	1	54	2	0,02
45	2	3.45	Male	Left	No	18	1	1	47	2	0,23
46	2	2.83	Female	Left	Yes	9	2	1	47	2	0,02
47	2	5.62	Male	Left	No	23	2	1	47	2	0,46
48	2	8.08	Male	Left	No	30	2	1	49	3	0,04
49	3	1.78	Male	NA	NA	NA	NA	NA	NA	NA	0,06
50	3	7.22	Male	NA	NA	NA	NA	NA	NA	NA	0,11
51	3	8.39	Male	NA	NA	NA	NA	NA	NA	NA	0,10
52	3	3.08	Male	NA	NA	NA	NA	NA	NA	NA	0,65
53	3	6.52	Female	NA	NA	NA	NA	NA	NA	NA	0,83
54	3	3.08	Male	NA	NA	NA	NA	NA	NA	NA	0,18
55	3	8.59	Female	NA	NA	NA	NA	NA	NA	NA	0,01
56	3	8.14	Male	NA	NA	NA	NA	NA	NA	NA	0,09
57	3	5.28	Female	NA	NA	NA	NA	NA	NA	NA	0,13
58	3	6.02	Male	NA	NA	NA	NA	NA	NA	NA	0,66
59	3	10.19	Male	NA	NA	NA	NA	NA	NA	NA	0,16
60	3	7.56	Female	NA	NA	NA	NA	NA	NA	NA	0,06
61	3	7.99	Male	NA	NA	NA	NA	NA	NA	NA	0,12
62	3	9.51	Male	NA	NA	NA	NA	NA	NA	NA	0,14
63	3	7.40	Male	NA	NA	NA	NA	NA	NA	NA	0,16
64	3	4.22	Female	NA	NA	NA	NA	NA	NA	NA	0,90
65	3	10.04	Male	NA	NA	NA	NA	NA	NA	NA	0,03
66	3	4.82	Female	NA	NA	NA	NA	NA	NA	NA	0,01
67	3	4.14	Male	NA	NA	NA	NA	NA	NA	NA	0,07
68	3	3.99	Male	NA	NA	NA	NA	NA	NA	NA	0,03
69	3	0.54	Female	NA	NA	NA	NA	NA	NA	NA	0,11
70	3	6.53	Male	NA	NA	NA	NA	NA	NA	NA	0,17
71	3	8.47	Female	NA	NA	NA	NA	NA	NA	NA	0,00
72	3	4.52	Male	NA	NA	NA	NA	NA	NA	NA	0,02
73	3	15.21	Male	NA	NA	NA	NA	NA	NA	NA	0,12
74	3	2.67	Female	NA	NA	NA	NA	NA	NA	NA	0,01

NA = not applicable

## DISCUSSION

The present report describes a novel application of [TIMP-2] [IGFBP7]/1000, known as Nephrocheck®, a rapid urine-based diagnostic method that has already been incorporated into clinical practice to identify an AKI biomarker within critical care because it is simple to collect and may be used sequentially or in a monitoring context. This is the first study to analyze the performance of urinary [TIMP-2] [IGFBP7]/1000 in the context of obstructive nephropathy.

The pathophysiology of the increases in urinary IGFPB-7 and TIMP-2 levels in AKI are not well understood. Johnson and Zager argued that two mechanisms are responsible for this: 1) a decrease in glomerular permeability and 2) injury-induced tubular cell release of constitutively expressed TIMP2 and IGFBP7 into the urine (15). The mechanism of obstructive nephropathy involves many factors, including local ischemia due to distention and increased intratubular pressure, leading to tubular cell injury. Markers of cell cycle arrest, such as TIMP-2 and IGFBP7, may signal that the renal epithelium has been stressed and confer a shutdown function but may still be able to recover without permanent injury.

Urinary biomarkers for kidney injury are divided into 3 groups: stress, damage, and dysfunction. Stress markers are preformed and do not require genetic transcription to be expressed; they reflect conditions that are potentially reversible, and cell damage may or may not occur. Damage markers to be expressed need alterations in the renal cellular metabolism or they are produced by immune system cells that infiltrate the renal system, reflecting renal injury processes. Dysfunction markers originate from the exfoliation of viable cells into the tubular lumen secondary to necrosis and cellular apoptosis of the renal tubules, which reflect sustained insults to the kidneys, leading to a persistent decrease in the GRF. Nephrocheck® is classified as a test of urinary stress markers (16).

For the aforementioned reasons, our group chose to assess this promising biomarker of kidney damage in a different clinical scenario (other than AKI). Obstructive uropathy has a chronic nature; therefore, this may have contributed to the failure to reach a good AUC to distinguish patients with UPJO who require surgical repair.

A cut-off value of 0.3 (ng/mL)2/1000 for Nephrocheck® was developed to assess the risk of AKI in adults (14). Meersch found an AUC of 0.85 when studying [TIMP-2] [IGFBP7]/1000 to predict the risk of AKI in 51 children undergoing cardiopulmonary bypass surgery. Interestingly, baseline levels were high (approximately 1.0ng/mL2/1000) in all children and decrease after surgery, with the best cut-off value to predict AKI at 4 hours of 0.7 (lower than the baseline value and higher than the recommended cut-off value of 0.3 for Nephrocheck®) (17). In a chronic scenario, such as obstructive nephropathy, [TIMP-2] [IGFBP7] likely behaves differently from that in AKI. The median concentration of all groups was below the cut-off value proposed to predict AKI in adults and children.

The expression of these markers increases when there is any insult to the renal tissue. We found that urinary [TIMP-2] [IGFBP7] was higher in patients with HN than in controls (groups 1 and 2 vs. controls, p=0.0021), indicating that noxious stimuli occur at any grade of HN.

It is interesting to note that all patients with values greater than 1.0ng/mL2/1000 had diffused cortical atrophy (Onen's grading score of 4) on US, denoting that patient with severe HN have an irreversible renal damage that radically changes the cellular metabolism (Figure-3).

After stabilization of the injury or resolution of the pathology that causes renal lesions, a decrease in biomarker expression was expected. However, after surgical clearance, the patients persisted with the same urinary concentration of [TIMP-2] [IGFBP7]. It is likely that some alterations in renal cellular metabolism occurs permanently and may justify this behavior.

## STUDY LIMITATIONS

Our study had some limitations. Nephrocheck® was developed to predict AKI in critically ill adult patients in the intensive care unit (18). Despite the interesting results predicting 30-day mortality in neonatal and pediatric patients with AKI, Nephrocheck® has not been widely used in daily pediatric clinical practice (19).

In our sample, the prevalence of obstructive HN in children older than 2 years was 58.3%, which is higher than that reported in the literature (15-25%) (20). Most prenatal HN cases resolve in a mean time of 30 months, and patients that require intervention will usually undergo surgery during this period (21). Our patients might have received a delayed diagnosis due to the complexity of the referral system of our public health system, which delayed the first visit to the specialist following the identification of HN (usually in prenatal period).

Future prospective studies are necessary to better define the diagnostic role and possible prognostic value of these parameters in the evaluation of HN in the neonatal population.

## CONCLUSIONS

In this initial evaluation of TIMP-2 and IGFBP-7 in pediatric patients with UPJO, despite showing a positive correlation according to the severity of obstruction, logistic regression after controlling for creatinine levels did not demonstrate that these cell cycle arrest biomarkers are reliable in predicting the need for surgical intervention in pediatric patients with UPJO.
